# The Peer Context of Dieting: The Relationship between Young Adults’ Dieting Frequency and Their Friends’ Weight-Related Characteristics

**DOI:** 10.3390/ijerph15122744

**Published:** 2018-12-05

**Authors:** Alexander Miething, Mikael Rostila, Christofer Edling, Jens Rydgren

**Affiliations:** 1 Department of Public Health Sciences, Stockholm University, SE-106 91 Stockholm, Sweden; mikael.rostila@su.se; 2 Department of Sociology, Lund University, SE-221 00 Lund, Sweden; christofer.edling@soc.lu.se; 3 Department of Sociology, Stockholm University, SE-106 91 Stockholm, Sweden; jens.rydgren@sociology.su.se

**Keywords:** dieting, ego-centric social networks, weight control behavior, early adulthood, Sweden

## Abstract

Previous research found that weight-related behaviors and body weight tends to be similar between individuals and peers. Rather little is known how different domains of weight-related behaviors co-evolve in peer networks. Hence, this study explores how young adults’ self-reported dieting relates to perceived body weight and weight control behaviors of their peers. A Swedish two-wave panel survey with ego-centric network data was analyzed with negative binomial regression models. Nineteen-year-old men and women in the first wave, and 23-year-olds in the follow-up sample were examined. Men at age 19 showed an increased dieting propensity when being exposed to underweight peers. Compared to men, women’s dieting at age 19 was more strongly related to their own body image concerns, and peers’ weight-related behaviors like physical exercising and unhealthy eating. The associations between dieting and peers’ weight-related characteristics for men and women deteriorated from age 19 to age 23. The findings suggest that women’s dieting—in comparison to dieting in men—is more strongly related to the peer context. The decrease in associations between men’s and women’s dieting and peers’ weight-related characteristics from age 19 to age 23 may reflect a weakened importance of the peer context in early adulthood.

## 1. Introduction

A substantial body of research has recurrently revealed peer similarity in terms of body weight and weight control strategies [1,2,3], but less is known about how different aspects of dieting and weight control behavior relate to each other. It is generally agreed that adolescents’ body images and appearance concerns are influenced by media exposure, parental behavior, and peer influences [4,5]. Peers appear to function as weight referents who provide information which is used by individuals to evaluate their own appearance, body weight, and weight control management [6]. Interactions with peers were shown primarily to amplify girls’ weight-related concerns and behaviors [7,8,9]. Despite the increasing expansion of dieting among younger men [10,11,12], research on males’ weight control behavior is more limited. Existing studies nevertheless suggest that peer interactions also relate to males’ weight-related behaviors [13]. Influences from peers are usually linked with gender-specific body change strategies—that is, increases of muscle mass in boys and attempts to lose weight in girls [14].

Peer clustering denotes the tendency of individuals to congregate into groups (e.g., social networks) based on a common set of objectives, ideas, and attributes. Social networks, therefore, represent a suitable frame for studying the spread and clustering of social behaviors, norms, and attitudes. They advance the transmission of social behaviors but also mark social boundaries as they constrain the diffusion of behaviors and attitudes to the members of a network [15]. Earlier research has shown that particularly problematic and unhealthy social behaviors tend to cluster and spread in social networks, including smoking [16,17], alcohol, and substance abuse [18], but also conditions like obesity [19]. Peer clustering in terms of BMI is more strongly pronounced in the higher BMI group [20]. In addition, excessive forms of dieting and weight control behavior, usually more common among girls, were shown to disseminate in peer networks [21].

“Homophily” denotes the similarity between individuals and peers, which is a central premise in social network research [22]. Adolescents in particular strive to identify desirable behaviors, attitudes, and norms to reduce the discrepancy between themselves and their reference group [23]. Individuals’ desire to connect with others who are similar to themselves constitutes a powerful force that structures social networks [24]. The processes that contribute to homophily are social influence and social selection [25]. It is still controversial and subject to debate which of these principles is more important in constituting homophily. Behaviors relevant to peer influence are often associated with higher status and popularity of peers [26]. By contrast, selection processes are the principal mechanisms for the diffusion of behaviors and characteristics which are subject to peer rejection and marginalization [1]. As exemplified in de la Haye et al. [1], marginalized overweight adolescents may feel safer when engaging with other overweight peers. In order to become socially contagious, behaviors and characteristics must be observable and relevant for peer group members [3,27,28]. Accordingly, clearly visible behaviors rather than attitudes tend to spread in peer networks [25]. In order to approximate the body weight of peers, individuals may exert different weight-control strategies (e.g., reduced intake of food, exercising, etc.). Therefore, individuals and peers also cluster by different domains of weight-control behaviors. The perception of peers’ weight-related behaviors may have a modeling function for individuals’ own body images and weight-control strategies [29,30]. In representing idealized norms, peers serve as “weight referents” that mirror and reinforce individuals’ weight-related motives and body image concerns [1]. When evaluating themselves against significant others, individuals tend to target reference persons who are better off. Such upward orientations reflect individuals’ strive for self-improvement, which is an important motive in social comparisons between peers [31,32,33].

Relatively little is known about how the relationships between individuals’ and peers’ weight-related behaviors develop from late adolescence to early adulthood. Whereas peer interactions and individuals’ susceptibility to peer pressure is particularly strong during adolescence, conformity to peers tends to decline in early adulthood [34]. This suggests that also individuals’ dieting becomes more independent from peers’ weight-related characteristics when entering early adulthood.

### Aims of the Study

Whereas previous research largely focused on behavioral similarities between network members, the present study explores how different dimensions of weight-related factors in individuals and their peers relate to each other and operate across the weight spectrum. Using ego-centric network data that are based on a sample of 19- and 23-year old individuals, the study examines the associations between young adults’ dieting propensity and available peers’ dieting-related behaviors. We investigate conditions and behaviors of peers that have previously been shown to predict obesity and body image concerns in younger individuals, but are comparably unexamined in regard of dieting among men and women in late adolescence and early adulthood. These factors (i.e., peers’ body weight, peers’ exercising, and peers’ eating) are relatively visible behaviors and attributes, and likely responsible for the transmission of body image concerns, eating behavior, and dieting in peer networks. Gender-specific analyses account for differences in intentions, intensity, and frequency in dieting between men and women [35,36,37]. As predispositions for dieting and weight control practices vary across the weight spectrum and by appearance concerns, individuals’ BMI and self-rated body image was included in the analysis. BMI (available for individuals but not for their peers) was also used to perform interaction analyses with peers’ dieting-related characteristics.

The study addresses the following research questions:

(1) How does men’s and women’s dieting relate to peers’ body weight and weight-related behaviors?
(2) Are associations between men’s and women’s dieting and peers’ weight-related characteristics dependent on men’s and women’s own BMI?
(3) Do associations between men’s and women’s dieting and peer weight-related characteristics depend on age (i.e., whether men and women are at age 19 or 23)?

## 2. Materials and Methods

The present study utilized a Swedish two-wave panel survey titled “Social Capital and Labor Market Integration.” The Ethical Review Board of Stockholm (2008/580-31) approved the study. Informed consent was obtained from each participant included in this study. The target sample was drawn from the Swedish population register and comprised 5695 Swedish-born citizens. All respondents were born in 1990. For the first wave in 2009, 2942 interviews were successfully conducted when most respondents were at age 19, which corresponds to a response rate of 51.7 percent. The second wave of the study was performed in 2013 when most respondents had turned 23 and comprised 2244 respondents, equivalent to a response rate of 39.4 percent. The primary reason for non-responses was the widespread use of unregistered pre-paid phones in this specific age-group, which impeded communication. The survey was performed as a telephone interview and conducted by Statistics Sweden (SCB). The study sample with complete information on the variables of interest encompassed 3123 individuals, clustered in 11,602 ego-alter pairs (dyads), with an average response rate of 38 percent. Of those, 4165 pairs were drawn from the first wave (Time 1), 4214 from both waves (Time 1 and Time 2), and 3223 from the second wave (Time 2).

The survey featured an ego-centric network set up with young adults answering questions about their own everyday lives, health behaviors, and social networks. Respondents (“egos”) were asked to name up to five persons (“alters”) they viewed as friends, and with whom they interact most frequently. These persons could include relatives, siblings, and romantic partners.

### 2.1. Outcome Variable

Counts of dieting represent the outcome variable and were derived from the question: “How often did you diet during the past twelve months?” The derived variable was coded as “0” for those who never diet and “1” or higher denoting how often a diet was undertaken. Values larger than “1”, for example, might indicate that respondents had been on several consecutive diets. Respondents reported at most 30 diets. While the used regression models utilized the actual number of reported diets (i.e., integer values from 0 to 30), the responses were grouped into four categories in the descriptive Table 1.

### 2.2. Peer Variables

A set of variables measured the characteristics and behaviors of named peers. These variables included respondents’ ratings of peers’ body weight; whether peers exercise less, as often, or more than the respondent; and whether peers eat healthily. Peers’ body weight rated by the respondents referred to three response alternatives: “Would you describe his/her body weight as underweight/normal/overweight?” Peers’ training behavior was evaluated with the question: “Would you say that he/she trains more often/as often/less often than you do?” Peers’ eating practices were assessed with the question: “Does he/she (alter #) usually eat healthy food?”

### 2.3. Individual Covariates

Respondents’ appearance concerns and BMI were considered as individual determinants for dieting. BMI was based on respondents’ self-reported body weight and height (BMI = kg/m^2^). Appearance concerns were based on their agreement with the statement, “I am happy with how I look” and included five response categories. The responses “strongly agree” and “agree” were coded as “0”, the categories “neither agree nor disagree”, “disagree”, and “strongly disagree” were coded as “1”.

### 2.4. Analytical Strategy

Negative binomial regression models were used to analyze the count outcome. The model also allows us to handle the exhibited over-dispersion in the used data. The data were arranged in ego-alter pairs and comprised up to five observations that correspond to ties between individuals and their peers. Therefore, the calculated incident rate ratios (IRR) refer to ego-alter pairs of ego-centric networks [38]. Deflated confidence intervals produced by the dyad-level analysis were corrected by using a robust cluster function that obtained individual-specific confidence intervals. The associations were fully adjusted for all shown variables. The subsequently performed plots with predictive margins show how associations between dieting and peer variables depend on individuals’ BMI. The analyses were performed in Stata (Version 15) (StataCorp LLC, College Station, TX, USA).

## 3. Results

Table 1 provides an overview of the distribution of individual and peer variables in the study sample. The figures show that 19-year-old women in the sample go on diets more than twice as often as 19-year-old men. Whereas the prevalence of dieting is increasing for men from age 19 to 23, the gender-differences in this practice diminish at age 23.

The mutually adjusted results from negative binomial regressions stratified by gender and panel wave are shown in Table 2. The peer network variables describe the associations between peers’ dieting practices and individuals’ dieting behavior. Compared to 19-year-old male respondents who interact with peers and whose body weight is perceived as normal by the respondent, male respondents who have an underweight peer in their network are more likely to diet (IRR = 1.81, 1.07–3.07). The association disappears at Time 2. Compared to women who engage with peers of the same body weight, those women who interact with overweight peers show an elevated dieting probability that is nearly significant at Time 1 (IRR = 1.52, 0.99–2.34). This association attenuates at Time 2. Instead, 23-year-old women with underweight peers in their network have 74% (1.12–2.70) higher probability of conducting diets. Peers training behavior reveals an association with women’s dieting at Time 1 only: 19-year-old women’s dieting propensity increases significantly when having peers who exercise more often (IRR = 1.96, 1.28–3.02) and less often (IRR = 1.73, 1.22–2.45) compared to women who have peers with training behavior similar to their own. Men’s dieting does not respond to the healthy or unhealthy eating practices of peers. By contrast, 19-year-old women who socialize with unhealthy eating peers show an altered dieting probability compared to women with healthily eating peers in their network (IRR = 1.51, 1.14–2.01). No association with (un)healthy eating was detected for 23-year-old women at Time 2. The covariates BMI and body appearance concerns show significant associations with dieting. Each one-point increment in BMI raises men’s probability of undertaking another diet by 31% (Time 2: 25%). For women the respective increase is 10% (Time 2: 11%). Women with high appearance concerns have a 2.3 times higher probability of dieting at Time 1, compared to a slightly lower probability at Time 2 (IRR = 2.1, 1.45–3.04).

**Figure 1:**
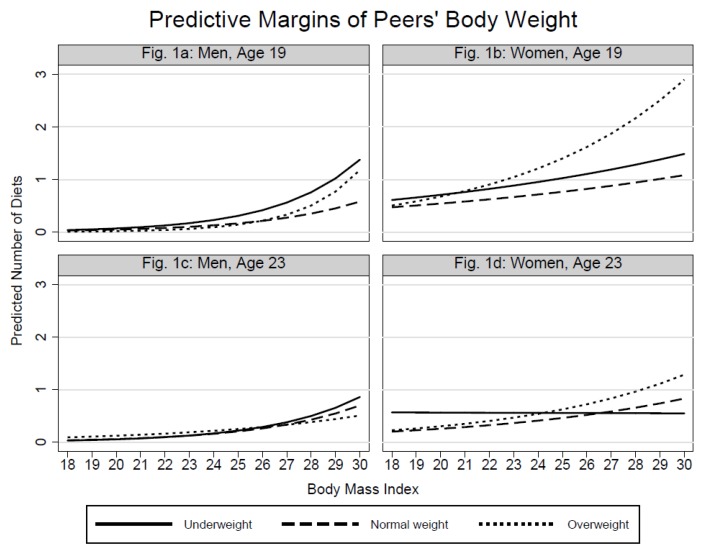
Predicted counts of dieting by peers’ body weight and individuals’ BMI.

**Figure 2:**
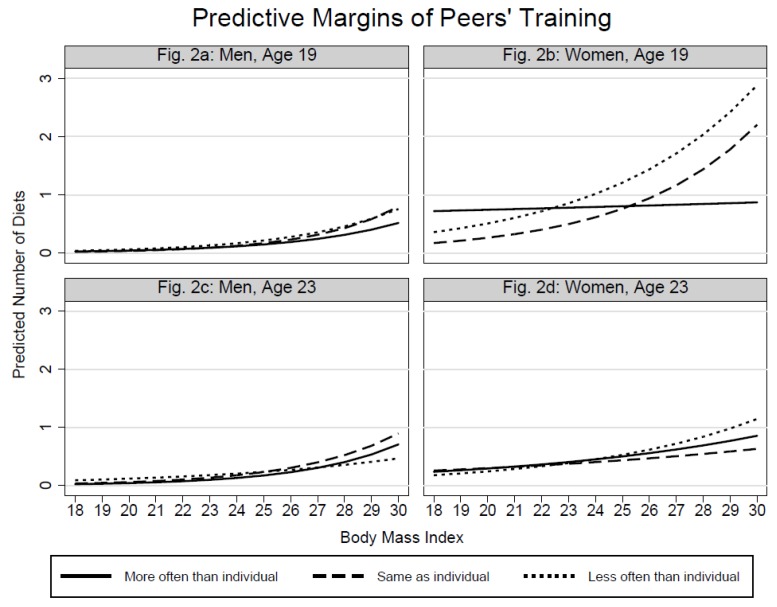
Predicted counts of dieting by peers’ training and individuals’ BMI.

**Figure 3:**
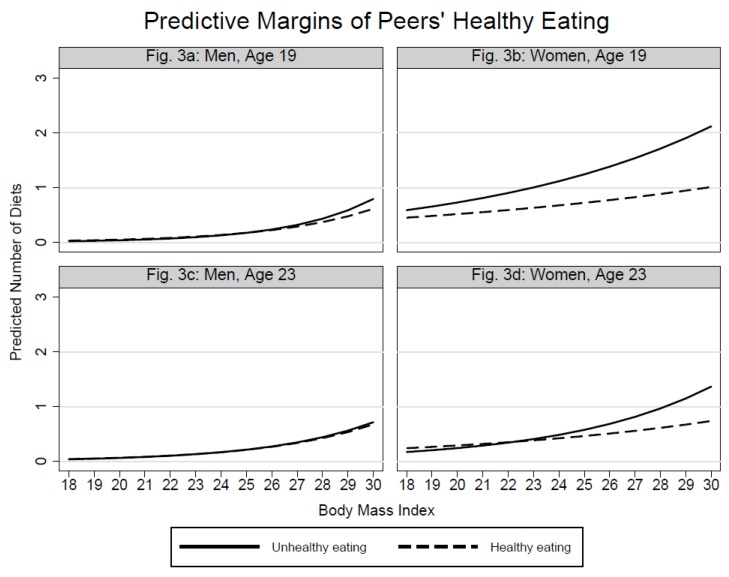
Predicted counts of dieting by peers’ eating practices and individuals’ BMI.

The plots with predictive margins (Figure 1, Figure 2, and Figure 3) were based on the previously shown regression models and intended to examine whether the associations between individuals’ dieting and BMI are dependent on peers’ dieting-related characteristics. Rather presenting trends than significance, the respective graphs show the predicted counts of individuals’ dieting by BMI at each level of peers’ body weight (Figure 1), peers’ training frequency (Figure 2), and peers’ eating practices (Figure 3).

Figure 1a shows that not only BMI, but also 19-year-old men’s exposure to underweight peers promotes dieting. In the higher BMI group, however, also contacts to overweight peers appear to increase men’s dieting. Figure 1b for 19-year-old women clarifies the V-shaped association between dieting and peers’ body weight: In the lower BMI spectrum, underweight and overweight peers give rise to a slightly higher probability of dieting. With increasing BMI the probability imposed by underweight peers does not rise as strongly as for contacts with overweight peers. The Figure 1c,d reflect the weaker associations between individuals’ dieting and peers’ body weight at Time 2. Figure 1d shows that the association between women’s dieting and peers’ body weight is dependent on women’s BMI. 23-year-old women with low BMI, who socialize with underweight peers, are more likely to diet compared to those who engage with normal- and overweight peers. With the exception of Figure 2b, the plots in Figure 2 confirm the null-findings from the analysis of main effects. For 19-year-old women the increase in the probability of dieting is strongest when they are exposed to peers who train less often. In the lower BMI spectrum, however, the dieting probability is higher for women whose peers train more often than the women themselves. Figure 3 shows that the predicted counts of dieting increase more for women who interact with peers who exhibit unhealthy eating practices. Again, the association is stronger at Time 1 compared to Time 2. The eating practices of peers do not show notable associations in the male sample.

## 4. Discussion

The study examined the associations between young adults’ frequency of dieting with their perceptions of peers’ body weight, exercise patterns, and eating behavior. The use of two-wave panel data allowed comparing these associations when respondents were at 19 and 23. Recognizing that body images differ between men and women [36], a gender-specific analysis was performed. Whereas previous network studies have mainly examined identical behaviors in individuals and their peers [39], the present approach studied individuals’ exposure to peers, i.e., how similar dieting-related aspects correlate between them. A particular focus was on the perceived discrepancies in individuals’ and peers’ body weight and training practices, and whether they predict individuals’ dieting propensity.

Distinct and partly opposed pattern in associations for men and women were detected. Women’s dieting revealed notable associations with all of the considered peer variables, whereas men’s dieting (at age 19) showed significant associations with peers’ body weight only. This suggests that young women in particular are sensitive to weight-related behaviors of peers in their network [40].

While the strength of associations with peers’ training and (unhealthy) eating tend to decrease from age 19 and 23, peers’ body weight still showed notable associations with dieting for 23-year-old women. A plausible explanation is that body weight comparisons matter for women’s dieting even after adolescence, whereas less visible behaviors (i.e., training and eating) become less important.

The marginal effects plots considered in addition the role of BMI and made clear that dieting women (but not men) in the higher BMI group tend to cluster with overweight peers [1,20]. This could point to a selection effect and indicate that social marginalization due to overweight pertains to younger women at age 19 [41]. Selection could also explain why 23-year-old dieting women with low BMI attach to underweight peers. It is plausible that dieting women (who are preoccupied with their own and others’ weight) to larger extent than men engage with peers who share a similar physical appearance and body weight. The significant association between 19-year-old women’s dieting and healthy eating of peers, but also the respective null-findings for men, confirm the notion that weight-related peer clustering occurs more often among women. Network members who share similar body weight characteristics are also more likely to engage in similar eating practices [42]. The diminishing associations from age 19 to 23 between individuals’ dieting and peers’ weight-related characteristics propose a deteriorating importance of the peer context for individuals’ weight-control behaviors. This finding is in line with previous research that has shown a decline in the susceptibility to peer pressure from adolescence to early adulthood [34].

Whereas appearance concerns and the peer variables revealed stronger associations in women compared to men, BMI correlated more strongly with dieting in men. This pattern is in line with the notion that peer discussions about weight-related issues reinforce female’s perceptions of body weight and dieting practices [43]. Likewise, cosmetic dieting (i.e., restricted eating without the objective need to lose weight) is more common among females, and likely a result of peer influence [40]. Men’s dieting in contrary seems to be more independent from the peer context, and to greater degree determined by individual conditions like BMI and overweight. The lower prevalence of dieting in men compared to women further reflects that this practice is less socially accepted among them [44]. For men in the lower BMI group, exercising (rather than counterproductive dieting) is a more effective strategy to come close to the idealized body weight and shape [43].

Dieting is a somewhat ambiguous measure as it reflects intentions and desires to lose weight, but also possibly comprises muscle-enhancing behaviors primarily in men [11]. Young adults’ dieting practices may also denote their efforts to become more similar either to peers’ body weight or peers’ weight control practices, or both. For example, the correlation of dieting with underweight peers—as shown for 19-year-old men and low-weight women at age 23—may reflect the diffusion of thinness ideals and practices like cosmetic dieting, while the correlation between dieting and overweight of peers—as demonstrated for women with high BMI—depicts the clustering of overweight network members [19]. As overweight is a highly observable attribute, overweight individuals are often targets of marginalization and social exclusion, which may reinforce the clustering of dieting individuals and overweight peers [1,3].

The fairly distinct findings for men and women in the present study may therefore be a result of different body ideals in men and women, but also arise from gender-specific clustering and social comparison processes between individuals and peers [45,46]. Affiliations with thinner peers seem to encourage men and particularly low-weight women to diet in order to maintain or lose weight. Conversely, female dieters in the higher BMI group do not benefit from low weight peers, which may reflect the demoralizing effects of upward orientations [33]. Overweight peers may elicit concerns in women about becoming overweight themselves and consequently motivate their dieting [47]. Whereas upward orientation (e.g., the affiliation with underweight peers) reveals the striving for self-improvement [31], downward orientation functions as a self-enhancing coping strategy, advancing individuals’ well-being and self-conceptions [32]. As argued elsewhere, self-esteem benefits from downward but not from upward comparisons [48]. People avoid upward social comparisons when they believe their ability to catch up with better-off others is hopeless [33].

### Strengths and Limitations

The study was based on unique ego-centric network data that included detailed information about respondents themselves and up to five named peers who are considered friends. The sample of 19 and 23-year-olds examined in this study represents an age group that is intensely engaged in peer interactions, sensitive to body image concerns, and susceptible to dieting. Despite these strengths, the study is subject to several limitations. It is not known how respondents conceive and interpret the interview question about dieting, and how intensively they diet—whether respondents skip meals, do not eat at all, or only reduce their intake of calories. In addition, the motivations and intentions for dieting remain unknown. Depending on these dispositions, dieting may compromise or enhance health. Furthermore, the survey question about healthy eating is rather vague. One might argue, however, that there is some rough common sense about healthy/unhealthy eating among respondents, and among serious dieters in particular.

Non-response, which resulted from the sampling procedure described earlier may have contributed to an under- or overestimation of associations. Because the data used were based on friendship-group members, respondents with problematic relationships may be underrepresented [3]. As the data used rely solely on information provided by the respondent, perception biases may have also affected the findings. Dietary and weight-related aspects may underlie a social desirability bias [49], resulting in misspecification of peers’ body weight and weight-related behaviors. Overweight individuals in particular have previously been shown to overestimate the body weight of peers [50,51]. The study design based on unbalanced two-wave panel data does not allow us to determine whether the associations are a result of social influence, social selection, or confounding from joint exposures. Therefore, it remains unclear whether individuals’ or peers’ behaviors are the structuring principle that imposes and enforces the identified correlations.

## 5. Conclusions

The aim of the study was to explore the associations between young adults’ dieting propensity and dieting-related practices of peers within friendship networks. The associations between individuals’ dieting and peers’ weight-related characteristics, particularly body weight and training, revealed distinct pattern and partly opposite associations for men and women. Women in the lower weight spectrum seem to affiliate with underweight others, while women in the higher BMI group orient downward to overweight peers. Independent of individuals’ body weight, underweight peers were identified as being important for men’s dieting propensity. The decreasing correlations of weight-related aspects from age 19 to 23 likely reflect a weakening susceptibility to the peer context during individuals’ transition from late adolescence to early adulthood. The important role of the peer group for younger individuals (i.e., adolescents) and the gender-specific motives for the uptake of dieting should be taken into account when tackling harmful weight control practices. Interventions may be more effective and multiplicative when not solely targeting individuals but cliques and peer networks as well.

## Figures and Tables

**Table 1:** Distribution of individual and peer variables.

	Time 1				Time 2			
	Men (age 19)		Women (age 19)		Men (age 23)		Women (age 23)	
Variables	Dyads	Percentage	Dyads	Percentage	Dyads	Percentage	Dyads	Percentage
**Outcome Variable**								
Individuals’ dieting (no. of attempts during the last 12 months)								
0 (not at all)	3028	91.9	2411	81.0	2351	86.3	2096	80.5
1 to 5 times	255	7.7	487	16.4	370	13.6	491	18.9
5 to 10 times	1	<0.1	26	0.9	2	0.1	9	0.4
10 to 30 times	11	0.3	53	1.8	2	0.1	9	0.4
**Peer Variables**								
Peers’ body weight								
underweight	202	6.1	231	7.8	143	5.3	200	7.7
normal	2810	85.3	2502	84.0	2329	85.5	2205	84.6
overweight	283	8.6	244	8.2	253	9.3	200	7.7
Peers’ training								
more often than individual	1341	40.7	1476	49.6	1159	42.5	1213	46.6
same as individual	928	28.2	865	29.1	708	26.0	702	27.0
less often than individual	1026	31.1	636	21.4	858	31.5	690	26.5
Peers’ eating								
healthy	2157	65.5	2184	73.4	1971	72.3	2096	80.5
unhealthy	1138	34.5	793	26.6	754	27.7	509	19.5
**Individual Variables**								
Individuals’ BMI (mean values with standard deviations in parentheses)	23.3 (3.0)		21.5 (2.9)		24.0 (3.0)		22.2 (3.3)	
Individuals’ appearance concerns								
happy with own look	2906	88.2	2222	74.6	2374	87.1	2053	78.8
not happy with own look	389	11.8	755	25.4	351	12.9	552	21.2
**Total**	3295	100.0	2977	100.0	2725	100.0	2605	100.0

**Table 2:** Incidence rate ratios (IRR) from negative binomial regression for counts of dieting (mutually adjusted for all covariates; 95% confidence intervals in parentheses).

Variables	Time 1				Time 2			
	Men (age 19)		Women (age 19)		Men (age 23)		Women (age 23)	
	IRR	CI 95%	IRR	CI 95%	IRR	CI 95%	IRR	CI 95%
Peers’ body weight								
underweight	1.81 *	(1.07–3.07)	1.28	(0.70–2.34)	1.07	(0.64–1.79)	1.74 *	(1.12–2.70)
normal weight	1		1		1		1	
overweight	0.85	(0.49–1.46)	1.52 †	(0.99–2.34)	1.26	(0.77–2.06)	1.33	(0.94–1.88)
Peers’ training								
more often than individual	0.91	(0.57–1.43)	1.96 **	(1.28–3.02)	0.81	(0.58–1.13)	1.07	(0.79–1.45)
same as individual	1		1		1		1	
less often than individual	1.33	(0.76–2.36)	1.73 **	(1.22–2.45)	1.08	(0.66–1.77)	1.01	(0.76–1.33)
Peers’ eating								
healthy	1		1		1		1	
unhealthy	0.98	(0.70–1.39)	1.51 **	(1.14–2.01)	1.01	(0.75–1.36)	1.05	(0.79–1.38)
Individuals’ BMI	1.31 **	(1.21–1.42)	1.10 **	(1.03–1.18)	1.25 **	(1.17–1.35)	1.11 **	(1.06–1.16)
Individuals’ appearance concerns								
happy with own look	1		1		1		1	
not happy with own look	1.66	(0.83–3.33)	2.31 **	(1.43–3.72)	0.81	(0.47–1.40)	2.10 **	(1.45–3.04)
**No. of dyads**	3295		2977		2725		2605	
**No. of individuals**	1272		1272		976		908	

† *p* < 0.10; * *p* < 0.05; ** *p* < 0.01.

## Availability of Data and Material

Because the data were linked to Swedish registers and subject to legal restrictions at Statistics Sweden, the full dataset used in this study is currently restricted to members of the LIFEINCON project. Specific requests for access to the survey part of the dataset should be addressed to the LIFEINCON PI at jens.rydgren@sociology.su.se.
